# Psychobiological and Psychosocial Responses of Nurse-Initiated Management of Antiretroviral Therapy–Trained Nurses Caring for People Living With HIV: Protocol for a Scoping Review

**DOI:** 10.2196/87562

**Published:** 2026-07-21

**Authors:** Phodzo Desire Tshelani, Muimeleli Munyadziwa, Lufuno Makhado

**Affiliations:** 1 Department of Public Health, Faculty of Health Sciences, University of Venda, 1 University Road, Thohoyandou, Limpopo, 0950, South Africa, 27 724184219

**Keywords:** health care workers' well-being, nurse-initiated management of antiretroviral therapy–trained nurses, NIMART-trained nurses, people living with HIV, psychobiological responses, psychosocial responses

## Abstract

**Background:**

Nurse-initiated management of antiretroviral therapy (NIMART) has expanded access to life-saving antiretroviral therapy and enhanced the decentralization of HIV care. The psychobiological and psychosocial responses of nurses implementing this strategy have been largely underexplored.

**Objective:**

This scoping review aims to systematically map the extent, nature, and characteristics of the existing literature on the psychobiological and psychosocial responses of NIMART-trained nurses providing care to people living with HIV.

**Methods:**

The review will be conducted in accordance with the Joanna Briggs Institute methodology and the population, concept, and context (PCC) framework, which will guide the development of the review question and define the eligibility criteria. A comprehensive search strategy will be used to identify empirical studies published between 2010 and 2026 across electronic databases (PubMed, SABINET, PsycInfo, CINAHL, Scopus, and Google Scholar). Two independent reviewers will screen the titles and abstracts to determine which studies are eligible. If there is any disagreement, a third reviewer will resolve it. Data will be analyzed using narrative synthesis and thematic mapping to identify patterns, knowledge gaps, and implications for future research, practice, and policy.

**Results:**

The review is expected to identify the scope, trends, and gaps in the literature on psychobiological and psychosocial responses among NIMART-trained nurses. The estimated outcomes will include a synthesis of existing evidence. The scoping review was initiated in July 2025, and is expected to be completed by the end of July 2026.

**Conclusions:**

This scoping review will provide an evidence-based understanding of the psychobiological and psychosocial responses of NIMART-trained nurses, highlighting implications for policy, practice, workforce support, and future research.

## Introduction

### Background

The global HIV epidemic remains a substantial challenge for health systems, especially in low- and middle-income countries, where resource constraints and workforce shortages continue to limit universal access to antiretroviral therapy (ART) [1-3]. To address these constraints, the nurse-initiated management of antiretroviral therapy (NIMART) course was adopted in the mid-2000s, based on the global strategy of task shifting, which was first endorsed by the World Health Organization (WHO) and partners to redistribute clinical tasks from physicians to other cadres in HIV care settings [3-5]. Implementation research in Africa confirms that one of the primary rationales for task shifting has been health worker shortages and the need to expand access to services [4,6,7].

Furthermore, recent evidence confirms that task shifting and task sharing have become foundational to health system resilience in sub-Saharan Africa, enabling more equitable service delivery amid ongoing workforce shortages [4,8]. Following this paradigm, South Africa adopted the NIMART policy nationally in 2010 to decentralize HIV care, enabling professional nurses at primary health care facilities to initiate and manage ART [6,9]. Research demonstrates that nurse-led ART initiation under NIMART has been both safe and effective in improving treatment coverage and patient outcomes, particularly in rural and underserved settings [6,10,11]. The expansion of NIMART has contributed significantly to South Africa’s success in scaling up ART initiation and retention across the HIV cascade [4,6].

However, the successful rollout of NIMART has not been without challenges. Evidence suggests that insufficient training, limited supervision, infrastructure deficits, and supply chain issues compromise nurses’ confidence and performance within NIMART settings [3,12]. A conceptual framework study conducted in the North West Province of South Africa highlighted the importance of continuous mentorship and organizational support in sustaining high-quality nurse-led HIV care [12]. Similarly, qualitative findings from Limpopo Province revealed that NIMART-trained nurses experience high workloads, burnout, and emotional distress linked to the dual management of HIV and noncommunicable diseases, particularly in resource-constrained clinics [13-15]. More recently, Ngcobo et al [16] identified operational barriers, including staff shortages, policy ambiguity, and limited managerial support, that continue to hinder the effective implementation of NIMART.

These studies collectively underline the increasing psychobiological and psychosocial burden placed on NIMART-trained nurses [6,12]. While task shifting has achieved impressive public health gains, it has also expanded the scope of professional nurses’ clinical and emotional responsibilities [17,18]. Global evidence shows that sustained exposure to heavy workloads and psychosocial stressors significantly increases the prevalence of burnout and job dissatisfaction among nurses [19-21]. These psychosocial challenges, if unaddressed, can erode workforce morale, reduce retention, and ultimately compromise the quality of HIV service delivery [22,23].

For this study, psychobiological responses will be defined as those that reflect both psychological and physiological processes related to stress and adaptation. In the review, a study will be classified as reporting psychobiological responses only if it includes at least one psychological outcome (eg, stress, burnout, anxiety, or depression) and at least one biological or physiological indicator (eg, cortisol levels, disturbed sleep, heart rate, blood pressure, or fatigue-related somatic symptoms) [24,25]. The psychosocial responses will be defined as the interaction between individual psychological experiences and the social and environmental context in which nurses work (eg, workplace relationships, organizational support, stigma, workload pressure, and coping mechanisms) [3,4].

Existing research has largely focused on patient outcomes, ART adherence, and health system efficiency, with limited synthesis of nurses’ internal experiences and coping mechanisms [6,12]. This scoping review protocol, therefore, aims to systematically map the available literature on the psychobiological and psychosocial responses of NIMART-trained nurses caring for people living with HIV.

### Identifying the Research Question

The primary research question is as follows: What are the psychobiological and psychosocial responses of NIMART-trained nurses providing care for people living with HIV globally between 2010 and 2026?

The subquestions are as follows:

- What are the psychobiological responses of NIMART-trained nurses providing care for people living with HIV?
- What are the psychosocial responses of NIMART-trained nurses providing care for people living with HIV?

## Methods

### Overview of the Methods for Conducting the Scoping Review

#### Study Design

This study will use a scoping review methodology, following the Joanna Briggs Institute (JBI) framework for scoping reviews, and will be reported using the PRISMA-ScR (Preferred Reporting Items for Systematic Reviews and Meta-Analyses Extension for Scoping Reviews) checklist [26]. The review is registered with the Open Science Framework.

The review will be designed using the population, concept, and context (PCC) framework, as recommended by JBI [27], to define the scope and inclusion criteria, as described in the following sections.

#### Participants

The review will include studies of NIMART-trained nurses aged 18 years and older who care for people living with HIV. It will examine research that explores their experiences, challenges, and responses to HIV treatment and support. Studies from different health care settings, including clinics and community programs, will be included. Both qualitative and quantitative studies that focus on NIMART-trained nurses will be reviewed.

#### Concept

The review will include studies focusing on the psychobiological and psychosocial responses of NIMART-trained nurses as they care for people living with HIV. Studies reporting both psychological and physiological measures will be classified as psychobiological, and studies reporting psychological outcomes within a social or environmental context will be classified as psychosocial. Studies reporting only social or organizational factors without psychological outcomes will be excluded. Studies that use interviews, surveys, or other research methods to explore these experiences will be considered. The aim will be to understand how their work affects them and what helps them cope.

#### Context

The review will include studies conducted in health care settings where NIMART-trained nurses provide HIV care, such as hospitals, clinics, and community health centers. Studies will not be limited to any one country, so research from across the world will be included. This will help provide a more comprehensive understanding of how the experiences of NIMART-trained nurses may vary across different settings. Studies from both rural and urban health care settings will also be included in the review.

#### Types of Evidence

Study characteristics will include study design and methodology (eg, qualitative, quantitative, or mixed methods), sample size, country, study setting, sample characteristics, and relevance to the review objectives. These characteristics will enable systematic recording during data extraction and will be summarized descriptively in both narrative and tabular form. The inclusion of studies will not depend on their methodological quality. Table 1 provides an overview of the eligibility criteria.

**Table 1.** Inclusion and exclusion criteria.

Category	Inclusion criteria	Exclusion criteria
Participants	NIMART^a^-trained nurses providing care for people living with HIV	Other health care professionals not trained in NIMART (eg, doctors, lay counselors, and social workers)
Concept	Studies examining psychobiological responses (psychological and physiological) and/or psychosocial responses (psychological outcomes within a social context)	Studies that do not address psychobiological and psychosocial responses or focus only on clinical patient outcomes
Context	Health care settings where NIMART-trained nurses provide HIV-related care (eg, clinics, hospitals, and community centers)	Non–health care or non–HIV-related contexts
Study characteristics	Empirical studies (quantitative, qualitative, and mixed methods studies; experimental and quasi-experimental designs) English-language publications Studies published between 2010 and 2026	Non-English publications or studies lacking empirical data due to insufficient translation Studies published before 2010

a NIMART: nurse-initiated management of antiretroviral therapy.

### Search Strategy

A 3-step search strategy will be used in accordance with the JBI methodology for scoping reviews [27]. The search process will ensure comprehensive coverage of both published and unpublished literature relevant to the review topic.

#### Step 1: Initial Search

An initial limited search of the PubMed, SABINET, PsycInfo, Scopus, CINAHL, and Google Scholar databases will be conducted to identify relevant literature and refine the search approach. The titles and abstracts of retrieved articles will be analyzed to identify indexing terms and synonyms related to the components of the review.

#### Step 2: Full Search

A comprehensive search will be conducted using all identified keywords and index terms across the following databases and sources: PubMed, SABINET, PsycInfo, Scopus, CINAHL, and Google Scholar. The search strategy will be customized for each database to account for differences in syntax and the use of Boolean operators. This step will ensure the inclusion of all relevant studies that meet the eligibility criteria.

#### Step 3: Searching Reference Lists and Gray Literature

The reference lists of all included studies will be manually reviewed to identify any additional relevant publications that may not have been captured through the database search.

### Study Selection

All retrieved records will be exported to EndNote (Clarivate), where duplicates will be removed. Two independent reviewers will screen the titles and abstracts for eligibility. They will also independently screen the full texts of the articles that meet the inclusion criteria. If any disagreement arises regarding article inclusion or exclusion, a third reviewer will resolve it. The study selection will be documented and presented using the PRISMA (Preferred Reporting Items for Systematic Reviews and Meta-Analyses) flow diagram [26].

### Data Extraction

The research team has developed a standardized data charting form for extracting data from the included studies. Extracted data will include the following study elements: authors; year the study was completed; country; study design; study setting; number of participants; participant demographic characteristics; psychobiological responses to burnout, including biological signs of stress (eg, cortisol levels, changes in heart rate or blood pressure, and physical symptoms) and psychological measures of distress (eg, stress, anxiety, depression, burnout, coping with stress, and perceived control over work-related tasks); psychosocial responses to work-related stress; coping strategies used by nurses to manage work-related stress; key findings; study limitations; and cultural factors. Data from each included study will be extracted by 2 reviewers, compared, and confirmed by a third reviewer.

## Results

### Overview

**Figure 1.**
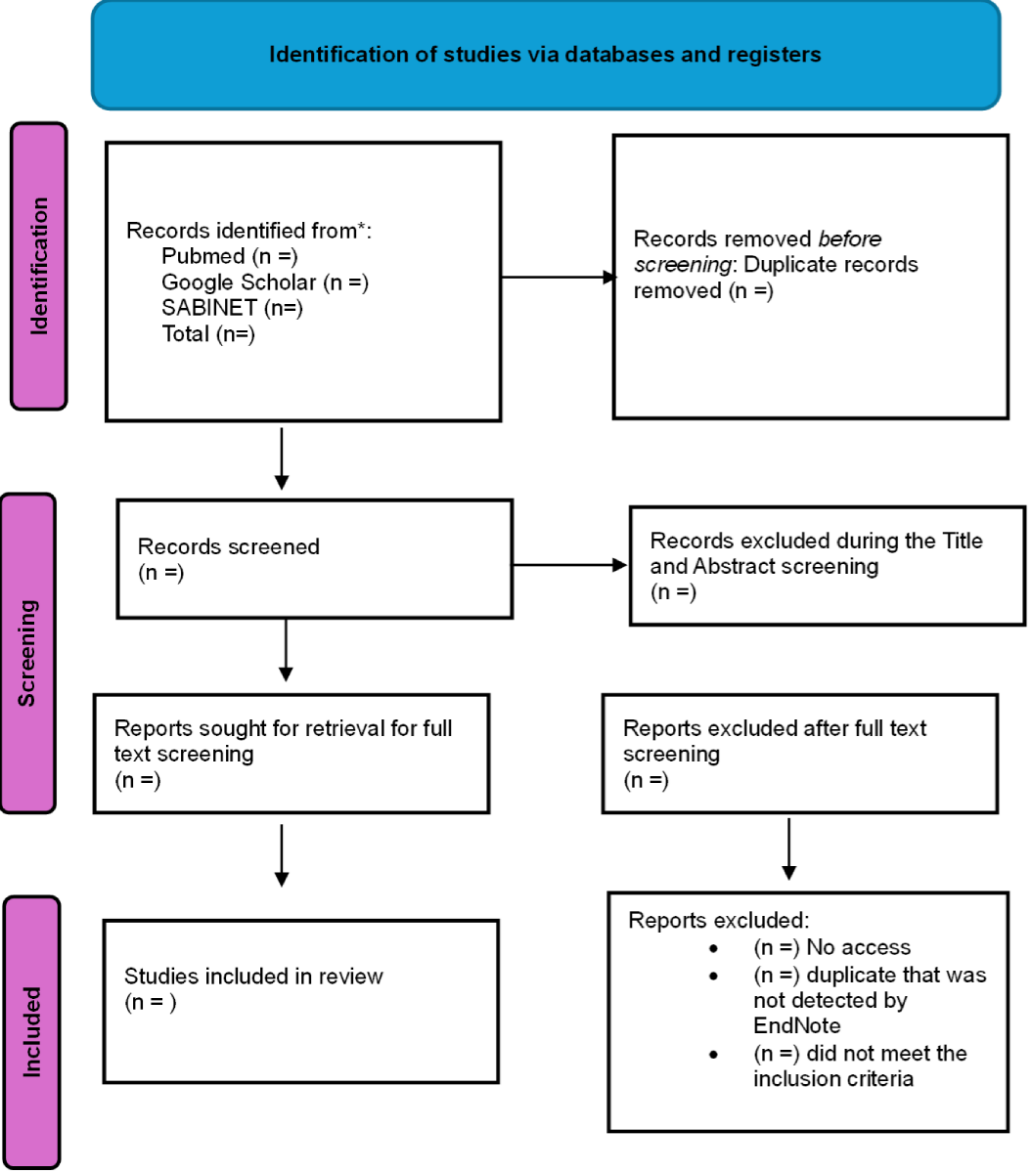
Preliminary PRISMA (Preferred Reporting Items for Systematic Reviews and Meta-Analyses) flow diagram.

Figure 1 presents the preliminary PRISMA flow diagram outlining the initial stages of the study selection process. The finalized diagram, showing the total number of studies identified, screened, and included, will be provided in the completed scoping review. The scoping review protocol was developed in July 2025 and is expected to be completed by the end of July 2026; the current study was not funded externally. The researchers plan to conduct database searches in May and June 2026, screen titles and abstracts in July 2026, and review full texts and extract data in August and September 2026, followed by data synthesis and analysis. The review team anticipates submitting the manuscript for publication in October 2026.

### Data Analysis and Presentation of Results

The data will be analyzed using both thematic mapping and narrative synthesis. These methods will facilitate the systematic categorization and summary of study findings to reveal key patterns and gaps in research describing the psychobiological and psychosocial responses of NIMART-trained nurses. It is anticipated that the review will identify significant gaps in support structures and highlight the need for targeted interventions to improve nurse well-being, retention, and, ultimately, the quality of HIV care. Completion of the scoping review is anticipated by October 2026.

## Discussion

### Principal Findings

This scoping review will document the body of research on the psychobiological and psychosocial responses of NIMART-trained nurses caring for people living with HIV. The anticipated findings of the review will provide a summary of the types of occupational stress, emotional burdens, coping methods, and workplace support systems reported in the literature regarding NIMART-trained nurses.

The review is expected to describe common patterns of how NIMART-trained nurses engaged in HIV service delivery report experiencing and documenting their psychological responses (stress, burnout, fatigue, and emotional distress). In addition, the review will explore psychosocial responses, such as workplace relationships, stigma, social support, and organizational factors, that affect nurses’ experience of delivering HIV care.

### Comparison With Previous Work

Previous studies demonstrate the psychosocial challenges facing the nurses who provide care for people living with HIV, including high burnout, emotional exhaustion, and depression due to heavy workloads and other pressures associated with providing care [28]. Additionally, the expansion of nursing roles resulting from the introduction of NIMART has been shown to increase nurses’ confidence when administering ART. This expansion has created operational challenges related to staff training, mentorship, and support systems, influenced by both professional and social responses [7,11,18]. A narrative review of NIMART over a decade further emphasizes the importance of factors that enable or prevent effective HIV care delivered by NIMART-trained nurses, including the quality of training, the level of supportive supervision, and the availability of human resources.

### Limitations

Potential limitations of this scoping review may arise from the decision to include only English-language literature, potentially excluding studies published in other languages. Additionally, there will be no formal assessment of the methodological quality of the studies included in this review, which may limit the conclusions regarding the strength of the available evidence.

### Future Research and Methods of Dissemination

This review may provide evidence to support future research and development of interventions that support the well-being of NIMART-trained nurses who care for people living with HIV. The results will be published in a peer-reviewed journal and presented at academic or public health conferences, nursing institutions that provide NIMART training, and health care facilities.

### Conclusions

In this scoping review, we will map the psychobiological and psychosocial responses faced by the NIMART-trained nurses. By identifying these responses, we seek to support the creation of necessary mechanisms to protect nurse well-being and ensure adequate care for individuals who rely on them for HIV care delivery.

## Supplementary material

10.2196/87562 Checklist 1 PRISMA checklist.

## Abbreviations

ART: antiretroviral therapy
JBI: Joanna Briggs Institute
NIMART: nurse-initiated management of antiretroviral therapy
PRISMA: Preferred Reporting Items for Systematic Reviews and Meta-Analyses
PRISMA-ScR: Preferred Reporting Items for Systematic Reviews and Meta-Analyses Extension for Scoping Reviews
WHO: World Health Organization
